# Baf45a Mediated Chromatin Remodeling Promotes Transcriptional Activation for Osteogenesis and Odontogenesis

**DOI:** 10.3389/fendo.2021.763392

**Published:** 2022-01-03

**Authors:** Theodore Busby, Yuechuan Chen, Tanner C. Godfrey, Mohammad Rehan, Benjamin J. Wildman, Caris M. Smith, Quamarul Hassan

**Affiliations:** RNA Biology and Epigenetics Laboratory, Department of Oral and Maxillofacial Surgery, School of Dentistry, University of Alabama, Birmingham, AL, United States

**Keywords:** Baf45a, Brg1/Smarca4, osteoblastogenesis, chromatin remodeling, PBAF complex, dentinogenesis

## Abstract

Chromatin remodeling, specifically the tissue-specific regulation in mineralized tissues, is an understudied avenue of gene regulation. Here we show that *Baf45a* and *Baf45d*, two *Baf45* homologs belong to ATPase-dependent SWI/SNF chromatin remodeling complex, preferentially expressed in osteoblasts and odontoblasts compared to *Baf45b* and *Baf45c*. Recently, biochemical studies revealed that BAF45A associates with Polybromo-associated BAF (PBAF) complex. However, the BAF45D subunit belongs to the polymorphic canonical BRG1-associated factor (cBAF) complex. Protein profiles of osteoblast and odontoblast differentiation uncovered a significant increase of BAF45A and PBAF subunits during early osteoblast and odontoblast maturation. Chromatin immunoprecipitation sequencing (ChIP-seq) during the bone marrow stromal cells (BMSCs) differentiation showed higher histone H3K9 and H3K27 acetylation modifications in the promoter of *Baf45a* and *Baf45d* and increased binding of bone and tooth specific transcription factor RUNX2. Overexpression of *Baf45a* in osteoblasts activates genes essential for the progression of osteoblast maturation and mineralization. Furthermore, *shRNA*-mediated knockdown of *Baf45a* in odontoblasts leads to markedly altered genes responsible for the proliferation, apoptosis, DNA repair, and modest decrease in dentinogenic marker gene expression. Assay for Transposase-Accessible Chromatin sequencing (ATAC-seq) assay in *Baf45a* knockout osteoblasts revealed a noticeable reduction in chromatin accessibility of osteoblast and odontoblast specific genes, along with transcription factor *Atf4* and *Klf4*. Craniofacial mesenchyme-specific loss of *Baf45a* modestly reduced the mineralization of the tooth and mandibular bone. These findings indicated that BAF45A-dependent mineralized tissue-specific chromatin remodeling through PBAF-RUNX2 crosstalk results in transcriptional activation is critical for early differentiation and matrix maturation of mineralized tissues.

## Introduction

During cellular differentiation from stem cells to progenitors to differentiated cells, must orchestrate a definite gene expression program to ensure accurate patterning and tissue specificity. This gene expression program is fundamentally regulated largely by tissue-specific epigenetic regulation that results in spatiotemporal gene expression (1, 2).

Chromatin regulation occurs through a series of crosstalk between cell-type-specific regulators and chromatin remodelers/modifiers. The BRG-/BRM-associated factors (BAF) chromatin remodeling complex is an ATPase-dependent epigenetic machine with affinity to bind and slide or eject nucleosomes (3). This process allows accessibility of lineage-specific transcriptional machinery enabling timely and appropriate control of gene expression.

In mammals, BAF factors (4, 5) are highly polymorphic and form complexes composed of a single central ATPase, either BRG1 (SMARCA4) or BRM1 (SMARCA2) (6). Similar to the BAF complex, a family of Polybromo-associated BAF (PBAF) complex has been identified as major tumor suppressors in several malignancies (7–9). Gatchalian et al. recently defined a smaller, non-canonical GBAF complex composed of bromodomain containing protein 9 (BRD9) and glioma tumor suppressor candidate region gene 1 (GLTSCR1) or its paralog GLTSCR1-like (GLTSCR1L) in mouse ESCs that is distinct from the canonical ESC BAF complex (esBAF). Further mechanistic insights demonstrated that BRD9 interacts with BRD4, which guides the recruitment of GBAF complexes to chromatin to maintain the transcriptional network of pluripotency (10).

Among four families of chromatin remodeling complex (SWI/SNF, CHD ISWI, and INO80), the SWI/SNF (switching defective/sucrose nonfermenting) family remodelers are composed of 8 to 15 BRG1 associated non-catalytic subunits. The catalytic ATPase includes an HSA (helicase-SANT), a post-HSA, and a C-terminal bromodomain. The non-catalytic subunits with BRG1 or BRM generate conserved, modular, or defining BAF complexes for cell and tissue-specific function. These complexes include BAF, PBAF, embryonic stem cell BAF (esBAF), neural progenitor BAF (npBAF), and neuron BAF (nBAF). This family has many activities that include sliding and ejecting nucleosomes at many loci. However, they lack roles in chromatin assembly (11, 12). Collectively the mammalian BAF (mSWI/SNF) and PBAF complexes are comprised eight bromodomains (six on PBRM1, one on either BRG or BRM, and one on BRD7), two PHD finger proteins (BAF45 subunits), two chromodomains (BAF155 and BAF170), and between seven and nine DNA binding domain. A ligand-activated *in vitro* transcription assay was used to biochemically identify the PBAF complex, which was included with specific subunits such as BAF180 (polybromo PBRM1), BAF200 (ARID2), BAF45A (PHF10), and BRD7. The name polybromo BAF (PBAF) came from the discovery that this complex contained polybromo (PBRM1 or BAF180) and BAF200 (ARID2), as well as BRG1 (but not BRM) and several other subunits of the BAF complex (9, 13). At a given time, the BAF complex consists of about 15 BAF subunits. Tissue-specific regulation by the BAF complex arises from the combinatorial assembly of the 29 genes that make up this 2-MDa protein complex (9). The BAF complex promotes gene expression around active promoters, enhancers, and tissue-specific enhancers. To date, many studies have been performed to characterize the roles of ubiquitous and tissue-specific BAF subunits/complexes at the biochemical and physiological levels. Specific examples include during neurogenesis (14), hematopoiesis (15), immune responses (16), in development and disease (17) and embryonic stem cell functions (18).

In multicellular organisms, plant homology factor 10/BRG1 associated factor 45A (PHF10/BAF45A) is a distinct subunit of the PBAF subfamily, which is among the three classes of mSWI/SNF complexes (BAF, PBAF, and ncBAF) (19–21). Cross-linking mass spectrometry on BRD7-and PHF10/BAF45A-bound complexes with stepwise modular assembly authenticated that PBAF complexes contain the same common BAF core module, ARID2, BRD7, and PHF10/BAF45A for tissue-specific combinatorial diversity (21). BAF45A possesses the double tandem PHD finger domain at its C-terminus. Besides BAF45A, similar domains have been identified in other BAF45 homologs; the tandem domains can function through lysine acetylation (H3K14ac) and methylation (H3K4me3) to control the activation of gene transcription in a cooperative and inter-dependent manner (22). The PHD domain can facilitate lysine acetylation at 14, K9, and K27 in histone 3 (H3K14, H3K9, and H3K27) to regulate chromatin for gene activation (23). Originally PHF10 was discovered in *Drosophila melanogaster*, and homozygous deletion resulted in lethality. Weak mutation led to decreased viability, reduced *yellow* gene transcription, and sterility (24–26). Conditional BAF45A homozygote null mice survived until embryonic day 19.5. Survived animals have impairment in the frequency of repopulating long-term hematopoietic stem cells and committed myeloid progenitors (27). BAF45A plays a crucial role in transitioning from proliferating neural stem/progenitor cells to post-mitotic neurons (28), nonredundant and specialized functions within the developing hemopoietic tissue (27), and transcriptional activation in myelogenesis (29). However, the precise mechanism of chromatin remodeling is not fully understood, and very little is known about the BAF complex function during the differentiation of mineralized tissue.

Bone formation occurs through a series of transcriptional and signaling events aligned with epigenetic rulings that take mesenchymal stem cells into mature osteoblasts, osteocytes, and chondrocytes. Mature osteoblasts are differentiated from committed osteoprogenitors and pre-osteoblasts to form the bone matrix. This matrix further mineralizes ultimately forming mineralized bone. During this time, a unique gene expression program is needed to grow and differentiate osteoblast cells, which must be tightly regulated in a tissue-specific manner. Much of what we know about the regulation of this gene program comes from our understanding of the bone gene transcriptional program and bone-tissue-specific signaling (30, 31). Furthermore, growing studies about the epigenetic regulation involved in osteogenesis primarily focus on histone post-translational modifications, miRNA, and non-coding RNAs (32–39). However, the tissue-specific mechanisms involved in the openness of the chromatin around active genes are understudied in osteogenesis.

Dentinogenesis occurs, similar to osteogenesis, during the maturation of neural crest cells, dental pulp cells, or pre-odontoblasts differentiated into mature, mineral depositing odontoblasts. These cells form pre-dentin before calcification and maturation. After maturation and calcification, pre-dentin actively is transformed into dentin, the central mineral portion of the tooth (40–42). Like osteoblasts, a highly regulated gene expression program is needed to grow and differentiate odontoblast cells in a tissue-specific manner (43). Little is known about the epigenetic regulation of the odontoblasts, with less being known about chromatin remodeling in either tissue type (44, 45).

Our lab’s recent preliminary findings have demonstrated that the PBAF subunit BAF45A is preserving epigenetic balance for osteogenesis (37). There are four BAF45 homologs, *Baf45(a-d)*. Baf45d is thought to be expressed ubiquitously and is part of the canonical BAF complex. *Baf45b* and *Baf45c* are known to regulate neurogenesis and belong to npBAF (19).

*Baf45a* is a member of the polybromo-BAF (PBAF) complex and is unique from the other BAF45 subunits because of its N-terminal SAY domain, which could potentially enable its transcriptional activity (27).

Here, we investigated the chromatin remodeling mechanism by which the *Baf45a* promotes tissue-specific gene expression for the maturation and mineralization of osteoblasts and odontoblasts. Deletion of *Baf45a* in osteoblasts altered the chromatin state around gene promoters and enhancers that are important for osteoblast and odontoblast maturation. Furthermore, we begin to dissect further the temporal expression of *Baf45a* with other members of the PBAF complex that specifically regulate the chromatin remodeling in the osteoblasts and odontoblasts. Overall, our findings indicate that remodeling of tissue-specific chromatin by *Baf45a* is required to understand the chromatin accessibility and gene activation of bone and tooth formation.

## Materials and Methods

### Generation of Baf45a Early Craniofacial Mesenchyme Specific Conditional Knockout Mice

We purchased *Baf45a*‐floxed (*Phf10/Baf45a^f/f^*) mice from The Jackson Laboratory (*Stock* Phf10tm1.1Grc/J, Stock No: 019480*)* containing *loxP* sites flanking exons 3 and 4 as previously described (27). To excise *Baf45a* exclusively in early craniofacial mesenchyme, *Baf45a^f/f^* mice were bred with paired related homeobox 1 promoter Cre mice (*Prrx1-Cre* transgenic mice, Stock No: 005584, from The Jackson laboratory) (33). The subsequent *Baf45a^wt/f;^ Prrx1-Cre* mice were bred to obtain WT (*Baf45a^wt/wt^, Prrx1-cre*), Baf45aKO (Baf45a*^f/f^*, Prrx1-cre) male (n=3) and female(n-3) mice. Mice were genotyped by polymerase chain reaction (PCR) on tail genomic DNA with Accuris™ Mammalian Genotyping Kit (Accuris, Edison, NJ, USA). The *Baf45a* wild‐type and floxed alleles and the Cre transgene were detected by PCR using the specific primers listed in the **Supplementary Table S1**. Eight-week-old mice were used to study tooth phenotype.

### Animal Care and Use Statement

Animals were used for this research in accordance with guidelines implemented by the Institutional Animal Care and Use Committee and Animal Resource Program at UAB. Mice were housed in a facility with 12/12 lighting in standard rodent cages with free access to rodent chow and water. Pups were euthanized by decapitation prior to calvarial isolation at post-natal day 2-5.

### Cell Culture

The culture and maintenance of osteo and odontoblast cells were carried out as described previously (44, 46–49). HEK-293T cells (ATCC, Manassas, VA, USA) were cultured in Dulbecco’s Modified Eagle Medium (Corning Cellgro, Manassas, VA, USA); MC3T3-E1 cells and primary rat calvarial osteoblasts were cultured in Minimum Essential Media – Alpha (α-MEM; Hyclone, Cytiva, Marlborough, MA, USA); OD-21 cells (ATCC, Manassas, VA) were cultured in DMEM. All media was supplemented with 10% fetal bovine serum (FBS), 2 mM L-glutamine, 100 U/mL penicillin, 100 µg/mL streptomycin. Osteogenic and odontogenic differentiation was induced with the addition of 50 µg/mL ascorbic acid and 3 – 5 mM β-glycerophosphate (βGP; Sigma Aldrich, St. Louis, MO, USA). Cells were refreshed with fresh differentiation media at every 48 hrs.

**Rationale for using various *in vitro* cellular models:** This study utilized multiple *in vitro* cellular models of osteoblast and odontoblast differentiation: murine bone marrow stromal cells (BMSCs); murine preosteoblast MC3T3-E1 cells; rat OD-21 odontoblast differentiation, and primary calvarial preosteoblast differentiation. The primary goal of our study is to present evidence of the role of BAF45 homologs in cell-type-specific differences in their expression and chromatin remodeling potentials across the mineralizing cells of different tissue origins. We assume that having different cell lines will help to complement experimental data and add additional layers of experimental evidence. The nuanced differences between cell lines also yield new insights in addition to strengthening our overall findings. BMSCs and OD-21 cells (dental pulp stem cells) have shown remarkable similarities in their transcriptomic profiles. However, differential gene expression might affect their capacities of osteogenesis. Studying both OD-21 and BMSCs allow for complimentary analysis while still recognizing critical differences in the odontogenic vs. osteogenic pathways. MC3T3-E1 cells represent preosteoblast cells – a cell line more committed to osteogenesis than BMSCs. Primary calvarial cells likewise represent preosteoblasts but as a primary cell source and have been used as an ideal cellular model for many years. This adds additional confidence to study Baf45a’s contribution to osteoblastic differentiation and functional activity. Interpreting the difference of cell differentiation stages and kinetic among different cell lines, we validate and authenticate the differentiation stages for MC3T3-E1, mouse calvarial osteoblasts, and BMSCs and OD-21 cells by analyzing gene markers for proliferation, differentiation, and osteoblastic activity, mineralization potential, and osteoclast activity using ALP staining. The stages are indicated as proliferation (day 0-3), matrix maturation (day 10-14), and mineralization (day 15-20).

### RNA Isolation & RT-qPCR

TRIzol reagent extraction (Invitrogen, NY, USA) followed by DNAse1-treatment was used to isolate total RNA according to the manufacturer protocol from MC3T3-E1 cells, mouse calvarial osteoblasts, and OD-21 cells during osteogenic differentiation. RNA from short hairpin RNA transfected (Control *shRNA* and *Baf45a-shRNA*) odontoblasts was extracted by Direct-zol RNA kits (Zymo Research, CA, USA). RNA quantity and quality were measured using the NanoDrop spectrophotometer (Thermo Fisher Scientific, Boston, MA, USA) and samples diluted to the same RNA concentration prior to cDNA synthesis using the Primescript RT Reagent Kit (TaKaRa, Mountain View, CA, USA). Luna Universal qPCR Master Mix (New England Biolabs, Ipswich, MA, USA) was utilized for all qPCR reactions according to the manufacturer’s protocol. Gene expression levels were normalized to U6 small RNA or Gapdh mRNA expression. Results were calculated using the ΔΔCT method and expressed as Log2 Fold Change ± SEM. Student’s t-tests where α = 0.05 were performed to compare experimental versus control groups.

### *Baf45a* Overexpression

The full-length *Baf45a* cDNA (1663 bp cDNA, 497 aa, transcription ID # ENSMUST00000024657.12) with 12 exons was synthesized from Life Technologies Corporation (Carlsbad, CA) and cloned in pCDH-EF1-MCS-T2A-GFP vector (https://www.systembio.com/pcdh-ef1-mcs-t2a-gfp-cloning-and-expression-lentivector). Control empty vector and *Baf45a* overexpression constructs at concentrations of 1-2 ug/well of a 6-well plate were transfected into MC3T3-E1 cells at ∼50% confluence using FuGENE 6 transfection reagent (Promega Corp.), following the manufacturer’s instructions, and harvested after 72 h for protein and RNA analysis.

### Chromatin Immunoprecipitation Sequencing (ChIP-Seq)

The ChIP-seq data was obtained with the gracious permission of authors of “Chromatin dynamics regulate mesenchymal stem cell lineage specification and differentiation to osteogenesis” (50) from GEO (Series GSE76074). Data was uploaded to IGV and qualitatively analyzed using area under curve measurement with Adobe Photoshop. Data was normalized based on gene length and scale and plotted on a three-dimensional axis with Origin Pro 2019.

According to the protocol (50) ChIP-seq was performed on BMSCs undergoing osteogenic differentiation for 0, 7, 14, and 21 days. Formaldehyde was used to crosslink DNA to histone proteins and bound transcription factors before BMSC (5 × 10^7^ MSCs/timepoint) lysis and sonication, which produced protein-bound DNA fragments ranging from 200 to 600 bp. Chromatins were prepared and sheared as previously described (50). Sheared chromatin was then immunoprecipitated with Runx2 antibody (M-70, Santa Cruz), H3K4me (ab8895, Abcam, Cambridge, MA, USA), H3K9me3 (Ab8898, Abcam), H3K9ac (39137, Active Motif), H3K27me3 (07-449, Millipore), H3K27ac (07-360, Millipore), or immunoglobulin G (IgG) (12-370, Millipore). Protein-DNA complexes were purified using Protein-G Dynabeads (Invitrogen). DNA libraries were sequenced on an Illumina Genome Analyzer II or Illumina HiSeq-1500. Base calls and sequence reads were generated by Illumina CASAVA or bcl2fastq software (version 1.8 and 1.8.4, respectively, Illumina). Two independent biological repeats of ChIP-Seq libraries were prepared for each time point. Two input libraries were prepared with sonicated DNA for each time point to compensate for a possible variation of chromatin structure during MSC differentiation. For ChIP qPCR, primer sequences corresponding to genomic regions were generated and evaluated by RT-qPCR on an Applied Biosystems Viia 7 thermocycler (Thermo-Fisher).

### Assay for Transposase Accessible Chromatin (ATAC) Sequencing

*Baf45a* wild type and knockout calvarial cells were assayed for chromatin accessibility using the Assay for Transposase Accessible Chromatin sequencing (ATAC-seq) technique as described by Buenrostro et al. (51). For inducible deletion of *Baf45a*, the tamoxifen-inducible ubiquitously expressing CAGGCre-ER™ (JAX stock #004682) (36) mouse was bread into the *Baf45a^f/f^* mouse line. Primary calvarial cells were subsequently isolated and either treated with 1µM 4-OH tamoxifen (72 hours, refreshed media daily) or with an equal volume of ethanol as a mock treatment and differentiated in osteogenic media for 3 and 10 days.

Cells were lysed in ice-cold lysis buffer (10mM Tris-HCl pH 7.4, 10mM NaCl, 3mM MgCl2, and 0.1% Igepal CA-630). Lysates were incubated with Tn5 transposase (Illumina Tagment DNA Enzyme and Buffer Kit, Illumina, San Diego, CA, USA) for 30 min at 37°C, followed by DNA purification using the MinElute PCR Purification kit (Qiagen, Germantown, MD, USA). Transposed DNA was amplified with barcoded Nextera primers (Illumina, San Diego, CA, USA) using the NEBNext High-Fidelity 2X PCR Master Mix (New England Biolabs, Ipswich, MA, USA). The barcoded DNA libraries were sequenced using the Illumina sequencing platform and analyzed by the Genomics Core Lab at the University of Alabama at Birmingham Heflin Center for Genomic Sciences (Birmingham, AL).

### MicroCT Analysis

*Baf45a* was deleted at embryonic day E9.5 using *Baf45a^f/f^* mice and Prrx1-Cre mice. For micro-CT analysis, 2-month-old male and female *Baf45a^f/f^* and WT mice were dissected, and the whole head was fixed in 70% ethanol. The bone volume of the whole molar, the outer enamel layer, the inner dentin layer, and the whole mandible was measured using a Scanco 40 µCT. Samples were placed in a 12 mm diameter sample holder and scanned at 12 µm resolution, 55kVp, 145 µA with 200ms integration time. Data were analyzed using the µCT Evaluation Program (v.6.5-2, Scanco Medical). MicroCT was performed at the Small Animal Imaging Shared Facility at the University of Alabama, Birmingham.

### Stable Transduction of shRNA in OD-21 Cells

pLKO.1-puro lentiviral backbone (Addgene, MA) was used to clone *shRNA* targeting exons 3 and 4 of *rnoBaf45a* mRNA (sequence: *rno shRNA Baf45a* F: 5’-CCG GAA GCG GAA ATA TCC AGA TTT ACT CGA GTA AAT CTG GAT ATT TCC GCT TTT TTT G-3’ and *rno shRNA Baf45a* R: 5’-AAT TCA AAA AAA GCG GAA ATA TCC AGA TTT ACT CGA GTA AAT CTG GAT ATT TCC GCT T-3’) to generate *Baf45a*-specific *shRNA*. The *shRNA* sequences are designed from the N-terminal SAY (Supporter of Activation of Yellow) domain encompassing exons 3 and 4. Structurally, this particular domain is not present in the other three BAF homologs. pLKO.1 empty vector was used as control. Viral particles were generated in HEK-293T cells by co-transfection of pLKO.1 shRNA plasmid [(52), Addgene plasmid #10878] with packaging vectors pCMVΔR-8.91 [(53), Addgene plasmid # 12263] and pMD2.G [(54), Addgene plasmid 12259] using polyethylenimine (PEI) (Polysciences). Forty-eight hours post-transfection, viral supernatants were collected, filtered through a 0.45 µm syringe filter, and added to OD-21 cells for spin infection followed by 24 hours incubation before adding fresh medium. The cells were grown on puromycin (2ug/ml) selected media for 72 hours. The gene expression changes due to *Baf45a* knockdown were studied by RNA-seq analysis.

### Library Generation and RNA Sequencing (RNA-Seq) Analysis

To examine the potential gene regulatory function of *Baf45a* during dentinogenesis, we performed high-throughput RNA sequencing. Briefly, total RNA was isolated from control, and *Baf45a* shRNA transfected OD21 cells using Direct-zol RNA MiniPrep kit protocol (Cat # R2052, ZYMO Research) and treated with DNase I (1u/ug of RNA) to remove the genomic DNA. Preparation of cDNA library generation and transcriptome sequencing was conducted by Novogene Co., LTD (Beijing, China). A total of 0.5-1 μg of RNA per sample was used as input material. Ribosomal RNA was removed using rRNA Removal Kit or poly(A) capture, and the rRNA-free total RNA was recovered *via* ethanol precipitation. Total RNA was fragmented to a specific size range for whole transcriptome or mRNA sequencing using RNAse III. Afterwards, sequencing libraries were generated using the rRNA-depleted fragmented RNA using a cDNA Library Prep Kit for Illumina following Novogene mRNA-seq services and recommendations. First-strand cDNA was synthesized using random hexamer primers and M-MuLV Reverse Transcriptase (RNase H minus). Subsequently, second-strand cDNA synthesis was performed using DNA polymerase I and RNase H. The size-selected, adaptor-ligated cDNA was amplified with High-Fidelity DNA polymerase, Universal PCR primers, and multiplex oligo adapters. The library quality was assessed on the Bioanalyzer system to perform sequencing. The correct adapters to capture the directionality of RNA in the resulting cDNA is important to maintain the RNA strand information and reduce the gene overlaps. RNA-seq Pair-end reads were aligned to the mouse genome (mm10). A reference genome index was built using Bowtie v.2.0.6 (55). Raw count data per gene was calculated using HTSeq (56). The raw count matrix was then used by DESeq2 (57) to quantify gene expression levels as normalized counts. Cuffdiff57 was used to detect differentially expressed genes between control shRNA and *Baf45a* shRNA (58). Transcripts with an adjusted P < 0.05 were considered differentially expressed. The differential expression analysis was performed by edgeR software and shown as a volcano plot. Gene Ontology (GO) functional categories were annotated and top significantly differentially expressed genes (DEG) enriched in apoptosis, DNA replication, and cell cycle were extracted and presented in a heatmap using Heatmap2 R package (59).

## Results

### Increased Histone H3K9 and H3K27 Acetylation and RUNX2 Occupancy Indicate *Baf45a* and *Baf45d* Promoters Are Highly Accessible During Induction of BMSCs to Osteoblast Differentiation

**Figure 1:**
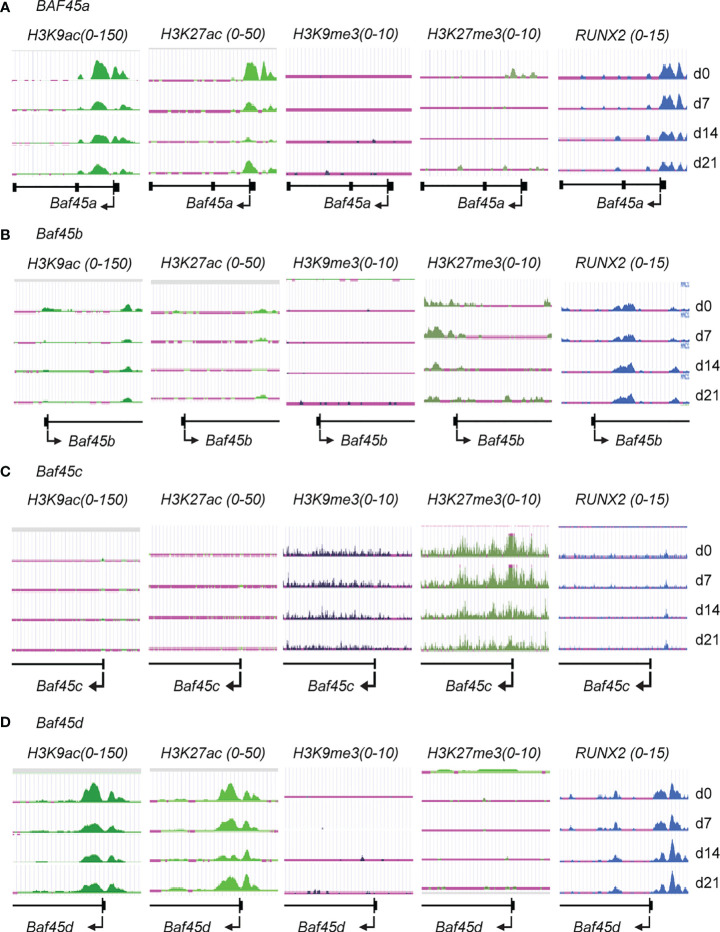
Histone H3 modifications and RUNX2 binding on the promoter regions indicate the accessibility of *Baf45* homolog promoters during Bone Marrow Stromal Cells (BMSCs) differentiation. Comparison of ChIP-Seq data acquired by Illumina sequencing. BMSCs were isolated from the bone marrow of mice, grown, and differentiated under osteogenic differentiation conditions, harvested at days 0, 7, 14, and 21, and ChIP-sequencing was performed. H3K27ac, H3K27me3, H3K9ac, H3K9me3, and RUNX2 antibodies were used to immunoprecipitate protein-DNA interactome **(A)** Genomic tracks display ChIP-seq data showing enrichment profiles for H3K27ac, H3K27me3, H3K9ac, H3K9me3, and RUNX2 at **(A)** *Baf45a*, **(B)** *Baf45b*, **(C)** *Baf45c* and **(D)** *Baf45d* gene promoters. Quantitative peaks representing active chromatin marks H3K9ac and H3K27ac and peaks representing repressive chromatin marks H3K9me3 and H3K27me3.

Studies previously suggested that *Baf45a* associated chromatin remodeling facilitates neurogenic differentiation (28). We currently identified that *Baf45a* remodeled chromatin implicate the transcriptional activation of osteoblast-specific genes (37). Lately, we found that osteoblast-specific deletion of *Baf45a* reduced bone formation *in vivo*. Originally, Baf45a was discovered as a target of miR-23a, and the knockdown of the miR-23a cluster has a high bone mass phenotype (manuscript in preparation/unpublished). Furthermore, we found that the bone tissue-specific RUNX2-BAF45A-EZH2 molecular epigenetic axis regulates osteogenesis *in vivo* (37, 38). These are the scientific premises behind choosing *Baf45a* to study further. However, the process of chromatin remodeling events and transcriptional activation mediated by *Baf45a* in osteoblast or odontoblast maturation and differentiation are not well-defined. We examined ChIP-seq data from primary bone marrow stromal cells (BMSCs) over a differentiation time course (50). BMSCs ChIP-seq analysis demonstrated that the genomic region around *Baf45a* and *Baf45d* gene promoters is active during osteogenic differentiation. High levels of the active chromatin marks H3K9ac and H3K27ac localized to the promoter regions of the *Baf45a* and *Baf45d* genes when compared with *Baf45b* and *Baf45c* (**Figures 1A, D** vs. **1B, C**). Because of the presence of the active chromatin modification marks, it is apparent that there would be a low or undetectable level of the repressive chromatin marks, including H3K27me3 and H3K9me3 around these genes. Furthermore, BMSCs ChIP-seq analysis with anti H3K9me3 and H3K27me3 showed very low occupancy levels at the promoter regions of *Baf45a* and *Baf45d* compared to *Baf45b* and *Baf45c* (**Figures 1A, D** vs. **1B, C**). Of note, *Baf45b* displayed moderate levels of both active (H3K9ac) and repressive (H3K27me3) chromatin marks in the gene body (**Figure 1B**), typically characteristic of a poised transcriptional state. The *Baf45c* promoter region displayed the highest and most pronounced marks of H3K27me3 modification and lacked any activating chromatin marks (**Figure 1C**). Evaluation of the RUNX2 binding on *Baf45* homolog promoters revealed a significant binding of RUNX2 to the *Baf45a* and the *Baf45d* promoters (**Figures 1A, D**). Conversely, little to no RUNX2 binding was observed on *Baf45b* and *Baf45c* promoters (**Figures 1B, C**).

When taken together, our results showed that increased *Baf45a* and *Baf45d* chromatin accessibility are implicated for BMSC’s commitment to the osteoblast lineage and reinforce osteogenic differentiation.

### *Baf45a* Promotes Osteogenic Differentiation

*Baf45a* is a subunit of the PBAF complex, while *Baf45d* is functionally complexed to the canonical BAF (cBAF) complexes (21, 60). Our current study will specifically focus on *Baf45a* to identify its function linked to osteo and dentinogenesis.

To study the function of *Baf45a* in osteogenic differentiation, we first analyzed the expression of *Baf45a* during MC3T3-E1 osteoblast differentiation. Additionally, we overexpressed *Baf45a* in MC3T3-E1 pre-osteoblasts to ascertain its effect on the osteoblast marker genes.

**Figure 2:**
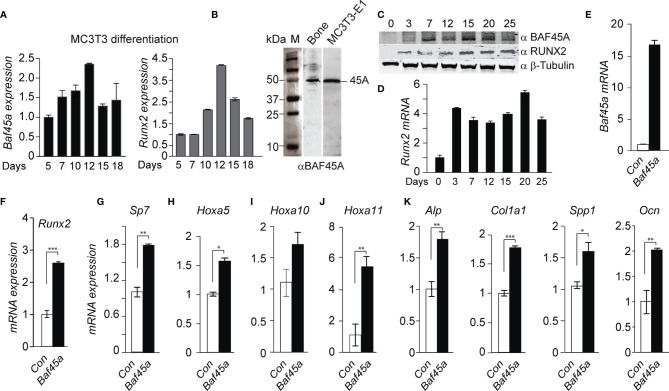
*Baf45a* expression levels induce gene expression profiles for osteoblast differentiation. **(A)** Relative expression levels of *Baf45a* (left panel) and Runx2 (right panel) were obtained by real-time RT-qPCR using DNase I treated total RNA isolated from murine preosteoblast MC3T3-E1 cells induced to differentiate for 18 days. *Gapdh* mRNA profile was used as an experimental control. **(B)** Representative Western blot analysis with anti-BAF45A antibody of lysates from the femur and MC3T3-E1 cells. Molecular weight markers were indicated in kDa. **(C)** Relative protein levels of BAF45A (upper panel) and RUNX2 (middle panel) were obtained by Western blot analysis using total cell lysate isolated from murine calvarial osteoblast cells induced to differentiate for 25 days. Beta Tubulin protein profile (lower panel) was used as loading control. **(D)** Relative expression levels of *Runx2* were obtained by real-time RT-qPCR using DNase I treated total RNA isolated from murine calvarial cells induced to differentiate for 25 days. **(E)** Overexpression of *Baf45a* in MC3T3-E1 osteoblast cells normalized to *Gapdh* after 72 hours by RT-qPCR. **(F–J)** Expressions of *Runx2*, *Sp7* (osterix) osterix, *Hoxa5*, *Hoxa10*, and *Hoxa11* were assessed by RT-qPCR and normalized to *Gapdh*. **(J)** Early markers *Alp* and *Col1A1* and **(K)** late markers *Spp1*(*Opn*), and *Ocn* were assayed. Statistical significance was determined by Student’s t-test (*P ≤ 0.05; **P ≤ 0.01; ***P ≤ 0.001 versus matched control). Gapdh expression was used as the control.

The *Baf45a* mRNA analysis by RT-qPCR demonstrated a temporal pattern of *Baf45a* expression during osteoblast differentiation, with a sharp increase in early osteoblast’s growth (day 5-7), followed by peak expression levels in mature osteoblasts (day 10-12) and decreased expression during the mineralization stage (day 15-18) (**Figure 2A**). Runx2 is a well-characterized bone-tissue-specific transcription factor. As a marker, the temporal increase of *Runx2* during osteoblast matrix maturation (day 10-12) and mineralization (day 15-20) supports the osteoblast differentiation program (**Figure 2A**, right panel). This temporal pattern of increased expression indicated that *Baf45a* remodeling is required to activate the osteoblast differentiation program.

Analysis of protein levels of BAF45A in long bone and osteoblast cells, using anti-BAF45A antibody (**Supplementary Table S3**, NovusBio: Cat# NBP2-19795; Lot# 42837) indicated a specific 45 kDa BAF45A protein in both mouse bone and MC3T3-E1 osteoblast cellular lysate (**Figure 2B**). Further western verified a similar temporal pattern of BAF45A protein during calvaria-derived osteoblast differentiation, with an increase in protein levels in mature osteoblasts (day 7-15) and maintained the level during the mineralization stage (day 20-25) (**Figure 2C**). We also immunodetected additional BAF45A species in our western analysis on osteoblast cells. In 2017, Tatarskiy et al. reported that PHF10/BAF45A, expressed as four ubiquitous isoforms, has different domain structures, and two of them (PHF10-S isoforms) lack C-terminal PHD domains, which enables their phosphorylation by CK-1, and is degraded by β-TrCP (61). Further studies are needed to uncover the role of these BAF45A isoforms in chromatin remodeling and tissue-specific gene expression. The protein (**Figure 2C**, middle panel) and mRNA (**Figure 2D**) profiles of the Runx2 gene were used as a marker of mouse calvarial osteoblast differentiation time course. With the observed increase in expression of *Baf45a* during osteoblast differentiation, we hypothesized that *Baf45a* overexpression would promote osteoblast maturation by activating bone marker genes through their chromatin remodeling. Cells were transfected with control (empty vector) and *Baf45a* overexpression construct and then allowed to differentiate for 72hrs. Compared with the control, *Baf45a* overexpression substantially increased the expression of *Baf45a*-encoded mRNA (**Figure 2E**). Analysis of osteogenic transcription factors revealed that Baf45a noticeably induced *Runx2*, *Sp7*, *Hoxa5, Hoxa10*, and *Hoxa11* expression from 1.5 to 6-fold to promote osteoblast differentiation (**Figures 2F–J**). Among bone-specific markers, *Baf45a* increased early markers, including alkaline phosphatase (*Alp*) and *Col1a1* (1.5-2.0-fold) (**Figure 2K**, left panel), as well as *Spp1* (osteopontin) and *Ocn* (*Bglap*), the late markers of osteoblast mineralization (**Figure 2K**, right panel). When taken together, these results revealed that *Baf45a* remodels osteoblast chromatin and promotes osteoblast differentiation through activating genes essential for osteoblast maturation and mineralization.

### *Baf45a* Subunit Is Necessary for Odontoblast Differentiation

**Figure 3:**
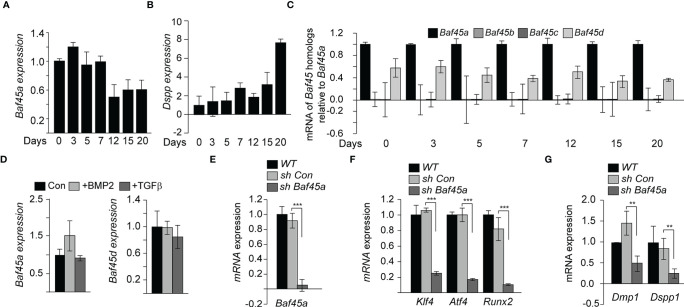
*Baf45a* functions promote odontoblast differentiation. **(A)** Relative expression levels of *Baf45a* were obtained by real-time RT-qPCR using total RNA isolated from murine odontoblast OD-21 cells induced to differentiate for 20 days. β-Actin cDNA was used as a loading control. **(B)** Real-time RT-qPCR analysis for the relative expression levels of *Dspp* using total RNA isolated from murine odontoblast OD-21 cells differentiated for 20 days. **(C)** Representative of mRNA analysis in triplicate (n=3) of *Baf45* homologs in OD-21 cells induced to differentiate for 20 days. **(D)** *Baf45a* and *Baf45d* responsiveness to BMP2 and TGFβ in OD-21 odontoblast cells. RT-qPCR analysis from OD-21 cells treated with BMP2 (100 ng/ml) and TGFβ (10 nM) at 80-90% confluence of culture and harvested 24 h post-treatment. **(E)** OD-21 cells were treated without *shRNA* (WT), control *shRNA* (*con sh*), and *shRNA* specific to *Baf45a* (*sh Baf45a*). After 72 hrs. post-treatment, RT-qPCR was performed on the RNA isolated from the harvested cells to assay the expression of *Baf45a*. *Gapdh* expression was used to normalize the relative gene expression. **(F)** Effect of *Baf45a shRNA* was assayed on the expression of Atf4, Klf4, and Runx2. **(G)** odontoblast differentiation markers *Dmp1* and *Dspp1* were assayed. *Gapdh* expression was assessed to normalize the relative expression. Statistical significance was determined by Student’s t-test (*P ≤ 0.05; **P ≤ 0.01; ***P ≤ 0.001 versus matched control).

Odontoblasts, the primary cell type in the tooth pulp, similarly deposit minerals to form detin as osteoblasts synthesize cortical and trabecular bone. We hypothesized that *Baf45a* mediated chromatin accessibility is critical for tooth-specific gene expression during odontoblast differentiation. To identify the role of *Baf45a* for odontoblast function, we studied rat odontoblast OD-21 cells to investigate differentiation. *Baf45a* mRNA was expressed at high levels during early differentiation and reduced by approximately 50% at day 12, corresponding to the mineral maturation period (**Figure 3A**). Dentin Sialophosphoprotein (*Dspp*) is a well-characterized dentin extracellular matrix phosphoprotein. The temporal increase of *Dspp* during OD-21 maturation and mineralization was used as a marker for odontoblast differentiation (**Figure 3B**). Relative to *Baf45a* expression, *Baf45b* and *Baf45c* were negligible (**Figure 3C**). *Baf45d* was also expressed at high levels during early odontoblast differentiation similar to *Baf45a* and consistently maintained later during mineralization stage (**Figure 3B**). These results indicate that like osteoblasts, *Baf45a* and *Baf45d* are preferred subunits of the PBAF and BAF complex in odontoblast cells. BMP2 treatment of OD-21 cells modestly (non-significantly) increased *Baf45a* expression (**Figure 3C**, left panel); however, no effect with BMP2 treatment in the expression of *Baf45d* was observed (**Figure 3D**, left and right panel). Transforming growth factor-β (TGF-β) is a secreted factor that modulates osteoprogenitors differentiation (62, 63). In odontoblasts, TGF-β1 signaling has a pivotal role in the transcriptional regulation of dentin sialophosphoprotein (DSPP) and dentin matrix protein 1 (DMP1) (64). It is unclear whether TGF-β directly modulates chromatin remodeler BAF45A and OD-21 induction for differentiation; therefore, we treated the cells with TGFβ to examine the effect of TGFβ on *Baf45a* expression. Treatment of OD-21 cells with TGF-β did not reduce expression of *Baf45a* nor *Baf45d* (**Figure 3D**, left and right panels).

To further investigate the role of *Baf45a* in odontoblasts, a short hairpin RNA (*shRNA*) was used to reduce the expression of *Baf45a* by nearly 90% (**Figure 3E**). We then hypothesized that *Baf45a* remodeling facilitates the expression of transcription factors *Klf4*, *Atf4*, and *Runx2*, affecting the expression of downstream odontoblast markers. With the shRNA mediated knockdown of *Baf45a, Klf4*, *Atf4*, and *Runx2*’s expression decreased significantly (**Figure 3F**). Analysis of odontogenic marker genes required for proper dentinogenesis showed decreased levels of *Dmp1* and *Dspp1* with loss of *Baf45a* (**Figure 3G**). This data suggests *Baf45a* is an essential subunit linked to odontogenic transcriptional induction and differentiation. Hence, when taken together, these findings suggest that similar to osteoblasts, *Baf45a* is important for odontoblast cells.

### BAF45A Led PBAF Complex Orchestrates Osteogenic Differentiation

**Figure 4:**
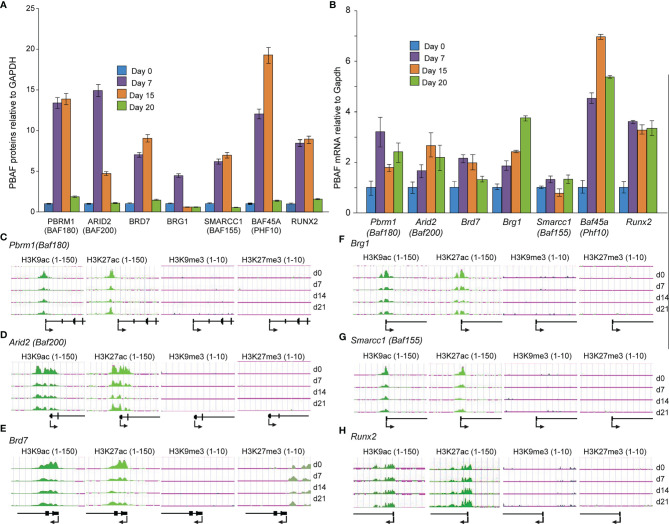
PBAF complex is critical for early differentiation and matrix maturation. **(A)** Quantitative Western blot analysis of PBAF factors from primary rat calvarial osteoblast cells induced to differentiate for 20 days. **(B)** Relative mRNA levels of PBAF complex, obtained by real-time RT-qPCR using DNase I treated total RNA isolated from murine calvarial osteoblast cells induced to differentiate for 20 days. **(C-H)** Histone H3K9 and H3K27 modifications on the promoter regions of PBAF complex members and *Runx2* during Bone Marrow Stromal Cells (BMSCs) differentiation. Illumina ChIP-seq genomic tracks at **(C)** *Pbrm1 (Baf180)*, **(D)** *Arid2 (Baf200)*, **(E)** *Brd7*, **(F)** *Brg1*, **(G)** *Smarcc1 (Baf155)* and **(H)** *Runx2* promoter regions. Quantitative peaks representing active chromatin marks H3K9ac and H3K27ac, as well as peaks representing repressive chromatin marks H3K9me3 and H3K27me3.

The functions and roles of PBAF in osteoblast and odontoblast differentiation remain largely undefined. In light of uncovering that BAF45A was a key factor of the PBAF complex in osteoblasts, we hypothesized that the BAF45A, including the members of the PBAF complex, are vital chromatin regulatory complex for determining chromatin accessibility for mineralized cells commitment and differentiation. To address our hypothesis, we first profiled the protein levels of the PBAF subunits during primary calvarial preosteoblast differentiation to address our hypothesis. We found a temporal protein profile during osteoblast differentiation, with moderate protein expression for all members of PBAF subunits between proliferation and early differentiation (day 0-5). This pattern was followed by significantly higher proteins starting at matrix maturation to early mineralization (day 7-15) and then decreased expression at the mineralization stage (day 20) (**Figure 4A**). Interestingly, the increase of BAF45A, PBRM1, and BAF200 proteins were noticeably higher, ranging from 5 to 17-fold and RUNX2 with 5-7-fold. When taken together, the expression of PBAF subunits during differentiation indicates a requirement of PBAF mediated remodeling for determined osteoblast-specific chromatin accessibility to facilitate the osteogenesis program. A temporal pattern of PBAF subunits and *Runx2* mRNA expression was observed during murine calvarial osteoblast differentiation, with significantly higher levels for all PBAF subunits and *Runx2* between early differentiation and matrix maturation stages (day 7-15), followed by lower (*Pbrm1, Baf200*, *Brd7* and *Baf45a*), steady (*Baf155* and *Runx2*) and higher (*Brg1*) mRNA levels in the mineralization (day 15-20) stage (**Figure 4B**). Taken together, these findings indicate that the chromatin remodeling event associated with PBAF functions may induce and maintain osteoblast/odontoblast differentiation. Next, we compared the osteoblast’s PBAF protein (**Figure 4A**) and mRNA profiles (**Figure 4B**) during differentiation to the chromatin modifications on PBAF subunit promoters (**Figures 4C–H**) in BMSCs to understand the epigenetic clues for the differentiation of two mineralized cells. BMSCs ChIP-seq analysis demonstrated that the chromatin modification landscape around PBAF subunits promoters consists of *Pbrm1 (Baf180), Arid2 (Baf200)*, *Brd7, Smarca4 (Brg1)*, and *Smarcc1 (Baf155)* displayed high levels of active H3K9ac and H3K27ac chromatin modifications (**Figures 4C–H** left two panels). Contrary to that, ChIP-seq analysis with anti H3K9me3 and H3K27me3 ChIP on the PBAF proteins mentioned above promoters showed low and undetectable levels (**Figures 4C–G**, right two panels). During differentiation, as expected, we found a reliable increase in H3K9 and H3K27 acetylation on the promoter region of the *Runx2* (**Figure 4G**). Hence, we posit that higher *Runx2* expression and RUNX2 binding to *Baf45a* promoter (**Figure 1D**) may promote a regulatory loop with PBAF subunits set the foundation for accessibility of chromatin and may activate transcription.

### BAF45A-PBAF Dependent Chromatin Accessibility Is Critical for the Expression of Mineralized Tissue Marker Genes

**Figure 5:**
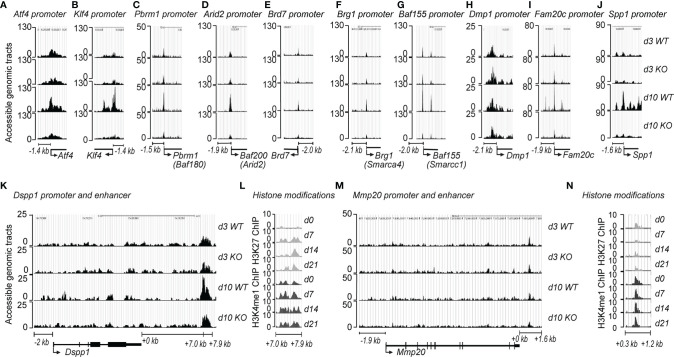
BAF45A-PBAF complex regulates chromatin accessibility and bone-specific gene transcriptional network. Baf45a floxed allele was deleted in primary calvarial osteoblasts *via* 4-hydroxytamoxifen inducible *CAG*-Cre genetic background. The cells were then subjected to osteogenic differentiation. Osteoblasts were harvested at day 3 and day 10 of differentiation. ATAC-seq was performed on WT and Baf45a knockout osteoblasts. **(A, B)** Chromatin accessibility at the gene loci of transcriptional factors *Atf4* and *Klf4*, **(C–G)** Genomic accessibility profiles of PBAF subunits, and tooth and bone-related genes **(H)** *Dmp1*, **(I)** *Fam20c*, **(J)** *Spp1*, **(K)** *Dspp1*, and *Mmp20* **(M)**. ChIP-sequencing analysis was performed in BMSCs using the anti-H3K27ac and anti-H3K4me1 antibodies to identify downstream enhancer regions of bone and tooth-related genes **(L)** *Dspp1*, **(N)** *Mmp20*. “Y-axis” indicates accessible genomic tracts, and the “X” axis denotes the genomic regions.

The binding of transcription factors (TFs) and RNA polymerase machinery to the promoter and enhancer regions of DNA is critical for regulating gene expression. BAF45A constitutes an essential subunit of the PBAF complex that promotes determined genomic accessibility for TFs and RNA Pol II for tissue specificity. To study how BAF45A mediated chromatin remodeling changes contribute to accessibility, we assessed chromatin accessibility in mouse calvarial osteoblasts (MOBs) derived from wild-type (WT) and *Baf45a* deleted mice during differentiation using ATAC-seq assays. We found that chromatin accessibility (promoter region surrounding -1.4 kb upstream) of the three crucial osteogenic transcription factors, *Runx2* (data not shown), *Klf4*, and *Atf4*, were significantly reduced in *Baf45a* knockout cells when compared to WT cells at day 10 (**Figures 5A, B**) (65–67). Little or modest change was observed on day 3. We assume that at day 3, the promoter accessibilities for differentiation are limited. Still, some cells would reflect accessibility for transcription initiation for the start of differentiation. Therefore, the BAF45A remodeling effect is modest. Therefore, this trend indicates that *Baf45a* deletion decreased promoter accessibility of these vital transcription factors required for induction and maturation of osteoblasts. The chromatin remodeling contributed by the BAF/PBAF complex in promoting osteoblast differentiation to activate bone tissue-specific gene expression is not clearly defined (68–70). With the idea that BAF45A has a strong association with the rest of the PBAF subunits for increased accessible chromatin, we next sought to test whether *Baf45a* deletion would cause subsequent changes in chromatin accessibility of PBAF complex members. We observed that chromatin accessibility of signature PBAF subunits, namely *Pbrm1, Arid2, Brd7, Brg1*, and *Baf155* (**Figures 5C–G**), showed non-significant changes at proliferation (day 3) compared to a noticeable high degree of diminished chromatin accessibilities at differentiation (day 10) at the promoter regions (encompassing - 1.4 to -2.1 kb upstream) in *Baf45a* deleted cells when compared to WT cells. We next assessed the promoter accessibility on osteoblast/odontoblast-specific promoters due to *Baf45a* deletion. We identified promoter regions for *Dmp1* (-2.1 kb upstream), *Fam20c* (-1.9 kb upstream), and *Spp1* (-1.6 kb upstream) are showing significantly diminished accessibility in *Baf45a* deleted cells compared to *WT* cells (**Figures 5H–J**). Interestingly, we did not observe any changes in the accessibility of the two essential marker genes, namely *Dspp1* and *Mmp20*, at their promoter regions. Therefore, we hypothesized that the cell-type-specificity of osteoblast/odontoblast cells might be controlled additionally by enhancer-like activity. We localized three distinct enhancer regions with accessibility changes when analyzed using ATAC sequencing of *WT* and *Baf45a* knockout cells. The regions located +7.0 to +7.9 kb downstream of *Dspp1* and +0.3 to +1.2 kb downstream of *Mmp20* genes (**Figures 5K, M**). These enhancers were further characterized using ChIP-sequencing with anti-H3K27ac and anti- H3K4me1 modifications during 21 days of BMSCs differentiation to support our ATAC results. These two enhancer-associated histone modifications were typical to define specific regions as enhancers contributing to gene expression changes. We observed high H3K27ac and H3K4me1 modifications surrounding the regions of *Dspp1* (+7-7.9 kb distal) and *Mmp20* (+0-1.6Kb distal) (**Figures 5L, N**) during differentiation. Taken together, our findings indicate that the PBAF complex, including BAF45A remodeling, is critical for osteoblast/odontoblast cell-type-specific transcriptional regulatory networks.

### Deletion of *Baf45a* in a Subset of Craniofacial Mesenchyme Modestly Reduced the Mineralization of the Tooth and Mandibular Bone

**Figure 6:**
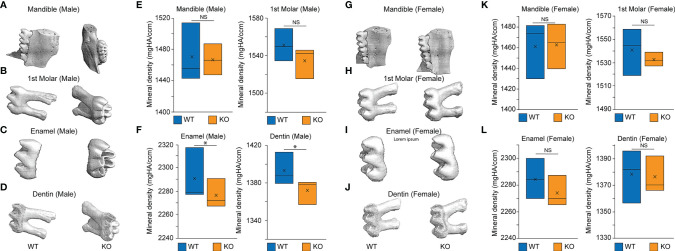
MicroCT of the mandible, first molar, enamel, and dentin in 2-month-old Baf45a conditional knockout mice compared to wild type. Baf45a^f/f^ was deleted at embryonic day E9.5 mating with Prrx1-Cre mice. **(A–F)** Homozygous *Baf45a^f/f^* (n = 3) and age-matched mice of 2 months were analyzed. Mice were euthanized, and teeth were extracted and stored in ethanol. The bone volume of the whole molar, the outer enamel layer, the inner dentin layer, and the whole mandible from *WT* and *Baf45a KO* male mice, **(G–L)** Bone volume of the whole molar, the outer enamel layer, the inner dentin layer, and the whole mandible from *WT* and *Baf45a KO* female mice. Males (n=3) and females (n=3) at 2-months littermates. Significance was determined using Student’s one-tailed t-test: *p < 0.05; NS (non significant). Mineral density (mgHA/ccm).

We hypothesized that *Baf45a* deletion would reduce tooth mineral density. Thus, we used *Prx1-Cre (Prrx1Cre*, Jackson Cat # 005584) transgenic mice to delete *BAf45a* in a subset of craniofacial mesenchyme (71). Molars from two-month-old male *Baf45a* deleted mice (n=3) had a modest reduction of tooth mineralization in the enamel and dentin layers (**Figures 6C, D, F**). However, a significant difference in the mandibular bone density of these same mice was not apparent (**Figures 6A, B, E**). In 2-month-old *Baf45a* deleted female mice, no difference in the mineral density in either the mandible or the molars (**Figures 6G–L**) were observed. Furthermore, a compensatory regulation from other BAF45 homologs might play a role in this regulation. Evidence of homolog switching comes from previous studies showing that the expression of BAF45A and BAF45D switch to BAF45B and BAF45C during development in early neurogenesis (28, 72). Thus, further mechanistic study of BAF45 homolog gene expression from dental pulp cells of *Prrx1-Cre; Baf45a^f/f^* mice would help us understand the compensatory mechanism.

### *shRNA* Knockdown of *Baf45a In Vitro* Regulates Odontoblast Growth, DNA Repair, and Apoptosis

**Figure 7:**
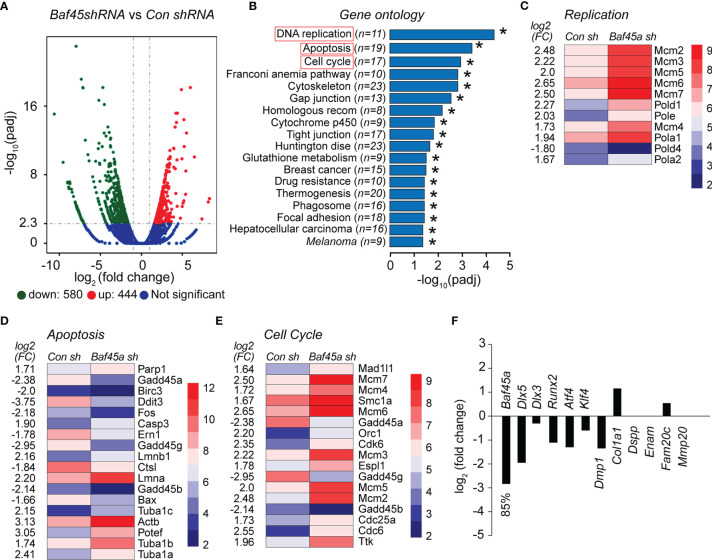
The shRNA mediated knockdown of *Baf45a* in odontoblast OD-21 cells affect growth, DNA repair, and apoptosis. **(A)** Volcano plot comparison of differential gene expression between control shRNA (non-specific) and Baf45a-specific shRNA. Red dots highlight upregulated, and green dots highlight downregulated differentially expressed genes with threshold FDR < 0.01 and Log2 Fold Change [≥ 1]. The blue zone indicates the number of transcripts that do not show significant differential expression. **(B)** Functional annotation based on Gene Ontology (GO) categorization. The horizontal bar plot represents differentially enriched genes linked to biological processes (FDR < 0.05) identified in control shRNA vs. Baf45a specific shRNA. *P < 0.05. **(C–E)**. Heatmap of differentially expressed genes belongs to DNA replication, apoptosis, and cell cycle. **(F)** The relative changes in the expression of osteoblast and odontoblast-specific transcription factors and markers genes due to *Baf45a* knockdown.

To evaluate the effect of *Baf45a* in odontoblast cells, we transduced OD-21 cells using lentiviral *Baf45a shRNA* and performed RNA-seq after 72hrs. Volcan Plot shows changes in mRNA expression between control (non-specific shRNA) and Baf45a shRNA (**Figure 7A**). Next, we compared the relative gene expression changes between control and *Baf45a*-specific *shRNA* transduced OD-21 cells using the RNA sequencing platform. Based on log2 fold change (FC), 444 upregulated (red dots) and 580 (green dots) downregulated mRNAs. Gene ontology (GO) functional annotations of significantly changed genes, using Database for Annotation Visualization and Integrated Discovery (DAVID) bioinformatics demonstrated the enrichment of pathways associated mainly with DNA replication, apoptosis, and cell cycle (**Figure 7B**). Amongst these genes, heatmap analysis found the highly conserved mini-chromosome maintenance proteins (MCM), component of the pre-replication complex (*Mcm2-7*), and DNA Polymerase subunits *α1* and *α2*, *δ1*, and *ϵ* were induced, but *Polδ4* was reduced in *Baf45a* depleted OD-21 cells (**Figure 7C**).

Further heatmap analysis of differentially expressed genes identified 18 factors associated with apoptosis and 17 factors linked to the cell cycle that were significantly altered in *Baf45a* deleted RNA compared with control shRNA (**Figures 7D, E**). Interestingly, we found that *Gadd45* homologs including *45a, 45b, and 45g* were significantly downregulated. These factors are associated with vital cellular processes, including growth control, maintenance of genomic stability, DNA repair, cell cycle control, and apoptosis (73, 74). We next hypothesized that *Baf45a* knockdown would affect early odontoblast differentiation. Therefore, we analyzed RNA-seq data for changes in the expression of odontoblast-specific transcription factors that induce differentiation and odontoblast differentiation marker genes. A significant knockdown of *Baf45a* mRNA decreased transcription factors *Dlx5, Dlx3, Runx2, Atf4*, and *Klf4*. Tooth matrix gene Dmp1 has been reduced; however, we did not observe any significant changes in the expression of *Col1a1*, *Dspp1*, *Enam*, *Fam20c*, and *Mmp20* resulting from *Baf45a* depletion (**Figure 7F**). Taken together, our data support that BAF45A promotes transcriptional activation in odontoblasts during differentiation, as we observed in osteoblasts.

## Discussion

Mineralized tissue phenotypes result from osteoblast or odontoblast cellular functions, controlled by bone and tooth-specific gene expression. Persistent upstream signals followed by regulatory interactions of chromatin remodelers, mediators, and insulators with aligned histone modifications mark in the promoter and enhancer DNA lead to the entry of transcriptional machinery. Most genetic and biochemical evidence indicates that BAF chromatin remodeling orchestrates the predetermined genome accessibility to control transcriptional gene activity in a tissue-specific manner (5–7, 9, 14–16). It is also evident that BAF remodelers’ function disruption resulted in inappropriate gene expression (17). The SWI/SNF chromatin remodeling complexes in mammals exist in three different forms: canonical BAF (cBAF), Polybromo BAF (PBAF), and non-canonical BAF (ncBAF).

Additionally, the PBAF complex is similar in function to the canonical BAF (cBAF) complexes with a few exceptions, one of which is incorporating BAF45A as a subunit in place of BAF45D. We focused our attention on PBAF from the perspective of BAF45A activity in mineralized tissue for specificity. Understanding the functions of chromatin remodeling complexes and the mechanisms underpinning their roles in bone and tooth formation are not defined. Here we studied the role of BAF45A, an essential subunit of the PBAF remodeling complex, in osteoblast and odontoblast growth, maturation, and differentiation.

### BAF45A and Osteoblast Chromatin Regulation

This study provides evidence that the function of BAF45A guided remodeling is vital to increase the chromatin potential for bone-tissue-specific gene expression. BAF45A, a member of the PBAF remodeling complex, was highly transcriptionally active, as shown by the deposition of H3K9 and H3K27 acetylation on the promoter. This deposition is indicating that BAF45A chromatin remodeling activity was necessary for BMSCs induction and osteogenic differentiation. Furthermore, increased RUNX2 occupancy with higher histone H3K9 and H3K27 acetylation in *Baf45a* promoter at days 0 to 7 demonstrated that osteoblast-specific RUNX2 promotes *Baf45a* transcription and hence mediates the bone-tissue-specific chromatin remodeling to induce and promote BMSCs for osteogenic differentiation. Overall, these findings indicate a collaborative crosstalk between BAF45A, PBAF complex members, and the transcription factor RUNX2 may exist to program the chromatin accessibility and transcriptional activation to initiate transcription of bone-specific genes necessary for bone formation. The relatively high expression of *Baf45a* up to osteoblast maturation (day 12) compared with the lower expression at mineralization (day 15-18) indicates that BAF45A mediated chromatin accessibility is essential to induce and accelerate osteoblast differentiation. BAF45A protein profile indicated a substantial amount of protein present in osteoblasts and bone tissue. Overexpression of *Baf45a* promotes the differentiation program of osteoblasts. Forced expression of *Baf45a* activates homeobox and tissue-specific transcription factors, early and late bone marker genes, and secreted phosphoprotein 1 necessary for bone formation. Thus, our research indicates that the BAF45A remodeled chromatins of transcription factors and marker genes in osteoblast cells may promote osteoblast transcriptional program at stages of differentiation. PBAF is a multi-subunit SWI/SNF chromatin remodeling complex, essential for cell proliferation and differentiation by directing eukaryotic gene transcription. In primary calvarial osteoblasts, we found that the increase of BAF45A, PBRM1, BAF200, BRD7, BRG1, and BAF155 proteins was noticeably higher with RUNX2 protein. This expression of PBAF proteins during differentiation indicates a requirement of PBAF mediated remodeling for determined osteoblast-specific chromatin accessibility. Further chromatin modification profiles indicate that BAF45A may act as a high-affinity H3 acetylation reader for histone H3 acetylation and, together with RUNX2, generates an epigenetic code for gene expression in osteoblast cell function and activity (75). Chromatin accessibility is vital for transcriptional machinery to the promoter and enhancer regions. BAF45A constitutes an essential subunit of the PBAF complex that promotes genomic accessibility for tissue-specificity, including bone and tooth. In *Baf45a* deleted mouse calvarial osteoblasts, where chromatin accessibility within 2kb upstream of osteoblast-specific transcription factors, marker genes, and PBAF factors were significantly diminished. Interestingly, we found that BAF45A deficiency is also reduced the accessibility of possible osteoblast-specific enhancers that may contribute to gene expression changes. These findings indicate that the BAF45A led PBAF remodeling forms accessible chromatins that are important to establish and maintain osteoblast cell-type and tissue-specificity.

### BAF45A and Odontoblast Chromatin Regulation

Our results in another mineralized cell, OD-21, showed BAF45A remodeled chromatin accessibility is critical for tooth-specific gene expression during odontoblast differentiation. We evidence that BAF45A and BAF45D are preferred subunits of the PBAF and BAF complex in odontoblast cells. BMP2 induction modestly increased *Baf45a*, but no effect was seen on *Baf45d* expression; interestingly, TGFβ signaling neither inhibits *Baf45a* expression nor *Baf45d.*

Additionally, knockdown of *Baf45a* significantly decreased mRNA of *Klf4, Atf4*, and *Runx2*, which are essentially required for activating tooth-specific genes transcription. *Baf45a* knockdown noticeably decreased *Dmp1* and *Dspp1* expression, critical for proper mineralization of dentin matrix and dentin. Hence, our findings suggest that, like osteoblasts, BAF45A mediates remodeling of odontoblast signature gene promoters directly or might be through the reduced transcriptional potential of RUNX2, ATF4, and KLF4 for odontoblast cell function. Hence, we believe that the regulation of tissue-specific mineralization *via* PBAF across these mineralized cells is identical. *Baf45a* deletion by *Prrx1-Cre*, at 2-months, showed subtle tooth abnormalities in males; however, in female mice, we did not observe any significant difference in the mineral density in either the mandible or the molars. Even though we noticed significant changes in gene expression and chromatin accessibility in multiple molecular biological approaches, the outcomes of such changes did not reflect tooth development and formation, particularly in females. One possibility might be due to limited Cre expression observed in female germline (oocytes). Additionally, the *Prrx1-Cre* effect might be affecting a wide variety of cells, including cells in cranial bone, chondrocranium, coronal suture, and lower jaw skeleton, which may not potentially commit to tooth formation. Similar minor changes were observed in molar dentin when Foster et al. deleted ALPL with *Prrx1-Cre* driver (76). Furthermore, as previously shown during neurogenesis, a compensatory regulation from other *Baf45* homologs might play a role in this regulation (28, 72). Gene ontology bioinformatics demonstrated the enrichment of pathways associated mainly with DNA replication, apoptosis, and the cell cycle. Analysis of differentially expressed genes identified 11, linked to DNA replication, 18 factors associated with apoptosis, and 17 factors related to cell cycle due to *Baf45a* knockdown. Furthermore, *Baf45a shRNA* decreased transcription factors *Dlx5, Dlx3, Runx2, Atf4*, and *Klf4*. Tooth matrix gene *Dmp1* has been reduced; however, we did not observe any significant changes in the expression of *Col1a1*, *Dspp, Enam, Fam20c*, and *Mmp20* resulting from *Baf45a* depletion. Hence, we concluded that BAF45A promotes transcriptional activation primarily by remodeling the promoters of genes that transcriptionally induce odontoblasts differentiation.

**Figure 8:**
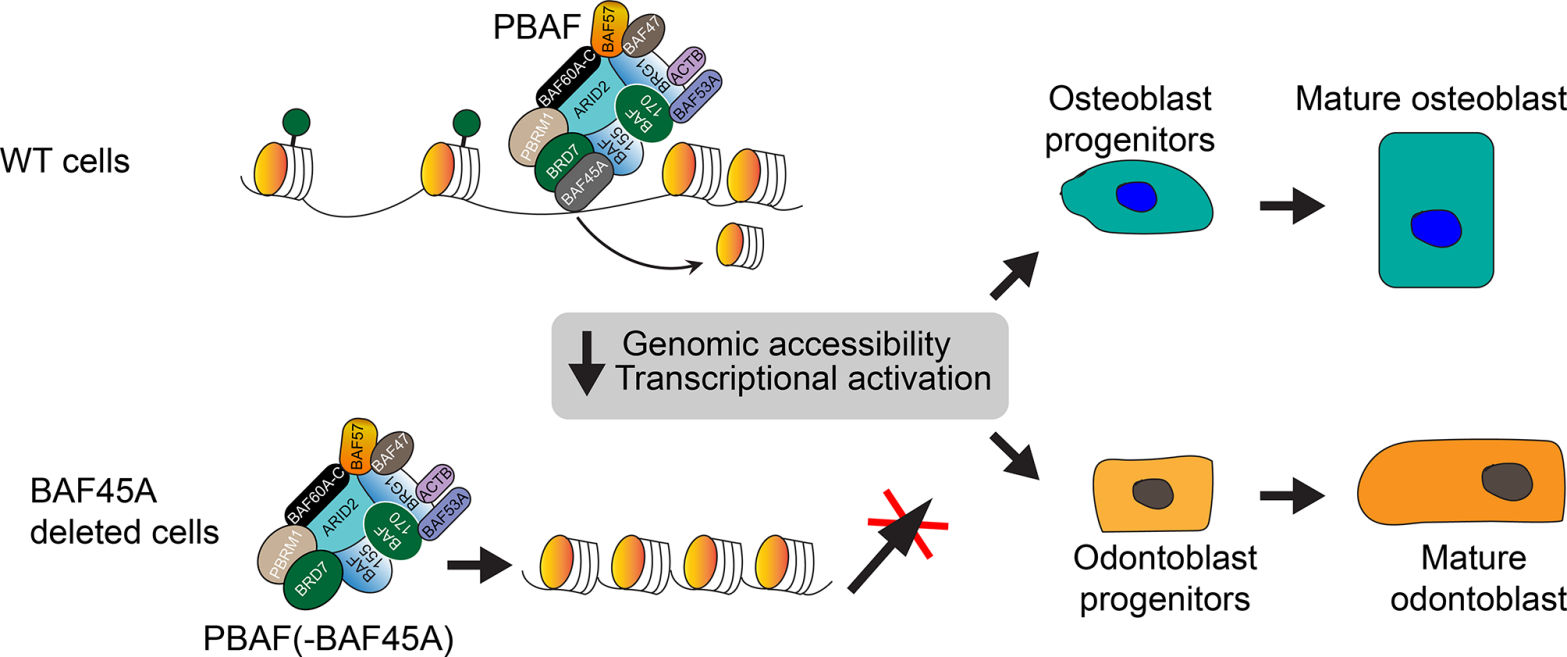
Working model: BAF45A led PBAF chromatin remodeling activity regulates transcriptional activation in mineralized cells. The model shows that the BAF45A is an integral subunit of the mammalian PBAF chromatin remodeling complex. BAF45a, with the help of PBRM1, ARID2, BRD7, and BAF155, evict nucleosome and promote chromatin accessibility to initiates transcription. Depletion, mutation, or complete knockout of Baf45a in osteoblasts and odontoblasts destabilizes or switches the PBAF complex composition. Recruitment of such defective PBAF complex inhibits relevant bone or tooth tissue-specific transcriptional events and gene expression. Finally, this inappropriate remodeling preventing the maturation of osteo or odonto-progenitor cells. Further biochemical assessment of BAF45A function with other PBAF subunits *in vivo* will help to understand chromatin remodeling in mineralized tissues better.

Our mechanistic model (**Figure 8**) shows that BAF45A evicts nucleosomes, so chromatin can be accessible to initiate transcription by PBAF remodeling. Depletion or ablation of *Baf45a* in osteoblasts and odontoblasts destabilizes or switches composition, resulting in defective PBAF complex formation, which leads to the inaccessibility of chromatin. Recruitment of such altered PBAF complex inhibits relevant bone or tooth tissue-specific transcriptional programs for gene expression. Thus, inappropriate remodeling preventing the replication, growth, and differentiation of osteo or odonto-progenitor cells. Further compositional assessment and rescue experiments for the members of the PBAF complex will better understand chromatin remodeling in mineralized tissues.

## Data Availability Statement

The BMSCs ChIP-sequencing data is deposited to the NCBI GEO database with the Accession number GSE76074 (https://www.ncbi.nlm.nih.gov/geo/query/acc.cgi?acc=GSE76074).

## Ethics Statement

The animal study was reviewed and approved by University of Alabama, IACUC.

## Author Contributions

TB designed the experiment, collected data, contributed to data analysis, and drafted the first version of the manuscript. YC performed and designed the experiment, troubleshoot the experiment, and contributed to data analysis. TG performed and analyzed ATAC-seq data and edited the manuscript. MR designed experiments, collected, and analyzed data. CS and BW performed experiments, analyzed data, and helped in manuscript editing. QH supervised the experimental design, reviewed data, supervised reproducibility of analysis, designed, and made figures, and supervised the manuscript writing for submission. All authors contributed to the article and approved the submitted version.

## Author Disclaimer

The content is solely the responsibility of the authors and does not necessarily represent the official views of the National Institutes of Health.

## Conflict of Interest

The authors declare that the research was conducted in the absence of any commercial or financial relationships that could be construed as a potential conflict of interest.

## Publisher’s Note

All claims expressed in this article are solely those of the authors and do not necessarily represent those of their affiliated organizations, or those of the publisher, the editors and the reviewers. Any product that may be evaluated in this article, or claim that may be made by its manufacturer, is not guaranteed or endorsed by the publisher.
