# Supplementary material for: Baf45a Mediated Chromatin Remodeling Promotes Transcriptional Activation for Osteogenesis and Odontogenesis
Source: Front Endocrinol (Lausanne). 2022 Jan 3;12:763392. doi: 10.3389/fendo.2021.763392 (PMC8762305; doi:10.3389/fendo.2021.763392)
Supplement: Supplementary file 1 [file Table_1.docx]

**Supplementary Table S1: Primers for real time quantitative PCR (RT-qPCR) assays**

| Gene Name | Primer Sequence |
| --- | --- |
| **BAF subunits (mouse)** | |
| *Baf155* | Forward: 5’-AAC GGC ATC AGG GGA CAT TT-3’  Reverse: 5’-GTA GAA ACC CCA GTG CAC CA-3’ |
| *Baf200* | Forward: 5’-TAA TGG CAA ACT CGA CGG GG-3’  Reverse: 5’-TCT TAA ACG GCG ACC CTC TG-3’ |
| *Baf180* | Forward: 5’-CCC CAC AGT CTA CCC CAA AGT-3’  Reverse: 5’-AAG AGT AGT CTG GGT GCT GG-3’ |
| *Baf45a* | Forward: 5’-GCG GAA ATA TCC AGA TTT AGA GC-3’  Reverse: 5’GTG TGC ACT GTG TTT CCG TG 3’ |
| *Baf45b* | Forward: 5’-CAG AAC CCG CTC AAG TCC CTT-3’  Reverse: 5’-AGT TGT TCT GGG CCA CTC C-3’ |
| *Baf45c* | Forward: 5’-GGA GAA CGA CGA CCA GCT AC-3’  Reverse: 5’-AGC TCC CAG CAT AAA TGG CA-3’ |
| *Baf45d* | Forward: 5’-TGC CTG TGA CAT TTG TGG AA-3’  Reverse: 5’-GGG GTC GGG AGT CTT CTT TG-3’ |
| *Brg1* | Forward: 5’-TCA AGG ATG ATG CCG AGG TG-3’  Reverse: 5’-CCT CGA TAG CCT TGA GCC AC-3’ |
| *Brd7* | Forward: 5’-CGA CCA TGA CAA ACA CAA GGA-3’  Reverse: 5’-GGT CTC GAT CCC GCT TCT TT-3’ |
| **Transcription factors (mouse)** | |
| *Runx2* | Forward: 5’-TGA TGA CAC TGC CAC CTC TG-3’  Reverse: 5’-GAT GAA ATG CCT GGG AAC TG-3’ |
| *Klf4* | Forward: 5’-CAC CCA CAC TTG TGA CTA TG-3’  Reverse: 5’-CTG GTC AGT TCA TCT GAG CG-3’ |
| *Atf4* | Forward: 5’-GCA CTT CAA ACC TCA TGG GT-3’  Reverse: 5’-CTC CAA CAT CCA ATC TGT CC-3’ |
| **Bone and tooth marker genes (mouse)** | |
| *Dspp1* | Forward: 5’-CAG GAC AAC CAG AAT CTC AG-3’  Reverse: 5’-CCA CTG AGC TTC CCA GAT TC-3’ |
| *Dmp1* | Forward: 5’-GAA AGC TCC GAA GAG AGG AC-3’  Reverse: 5’-CAC TCT TAG AGA GAC CAC CAG-3’ |
| *Col1a1* | Forward: 5’-CTT AGC TGC CTG GTT CTT CG-3’  Reverse: 5’-CCT TCT GGG ACC TCA AAT CA-3’ |
| *Enam*: S1 | Forward: 5’-GAA CAT CCC TCC ATA GCC TC-3’  Reverse: 5’-CAT GAC CAA GAG GCA AGT TGG-3’ |
| *Enam*: S2 | Forward: 5’-GAA GGA AGG AAC AAG CTG AAC-3’  Reverse: 5’-CAG GAC TGT GTG TGA GAT GAG-3’ |
| **Control genes (mouse)** | |
| *U6* | Forward: 5’-CGC TTC GGC AGC ACA TAT AC-3’  Reverse: 5’-AAA ATA TGG AAC GCT TCA CGA-3’ |
| *Gapdh* | Forward: 5’-AGG TCG GTG TGA ACG GAT TTG-3’  Reverse: 5’-TGT AGA CCA TGT AGT TGA GGT CA-3’ |
