# Supplementary material for: Baf45a Mediated Chromatin Remodeling Promotes Transcriptional Activation for Osteogenesis and Odontogenesis
Source: Front Endocrinol (Lausanne). 2022 Jan 3;12:763392. doi: 10.3389/fendo.2021.763392 (PMC8762305; doi:10.3389/fendo.2021.763392)
Supplement: Supplementary file 2 [file Table_2.docx]

**Supplementary Table 2: KLF4, ATF4 and RUNX2 binding motifs in**

**bone/tooth-specific promoters**

| **Transcription Factors** | **Gene Promoters and Position** |
| --- | --- |
| KLF4 (CACCC or GGGTG) | - *Dspp1*: -114 to -118 - *Dmp1*: -66 to -70, -130 to -134, and -822 to-826 - *Enam*: -1646 to - 1650 - *Mmp20*: +100, -11, -1026, -1290, -1662 - *Fam20c*: -60, -100, -592, -668, -789, -800, -1000, -1240, -1950 - Spp1: -894 |
| ATF4 (GTGACGT**RM** or **MR**ACGTCAC) M=A/G; R=A/C | - *Dspp*1: -1096 - *Spp1*: +86 |
| RUNX2 (TGTGGT or ACCACA) | - *Dspp1*: -626 - *Dmp1*: -373, -966 - *Enam*: -1332 - *Spp1*: -635 |
