# Supplementary material for: Baf45a Mediated Chromatin Remodeling Promotes Transcriptional Activation for Osteogenesis and Odontogenesis
Source: Front Endocrinol (Lausanne). 2022 Jan 3;12:763392. doi: 10.3389/fendo.2021.763392 (PMC8762305; doi:10.3389/fendo.2021.763392)
Supplement: Supplementary file 3 [file Table_3.docx]

| **PBAF Antibodies** | **Supplier** | **Catalog number** | **Lot number** | **Host** | **Dilution used** | **Secondary antibody used** |
| --- | --- | --- | --- | --- | --- | --- |
| **BAF180** | Millipore-Sigma | ABE70 | 3087162 | Rabbit polyclonal | 1:1000 | Anti-Rabbit 800 |
| **BAF155** | Santa Cruz Biotech  Cell Signaling Tech | sc-32763  11956 | 10718  2 | Mouse monoclonal  Rabbit monoclonal | 1:500  1:1000 | Anti-Mouse 680  Anti-Rabbit 800 |
| **BAF200** | Novus Biologicals: Cat. No; | NBP1-26615 | 19795 | Rabbit polyclonal | 1:500 | Anti-Rabbit 800 |
| **BAF45A** | Novus Biologicals | NBP2-19795 | 42837 | Rabbit polyclonal | 1:1000 | Anti-Rabbit 800 |
| **BAF45A** | Invitrogen | PA5-30678 | SL2491286C | Rabbit polyclonal | 1:1000 | Anti-Rabbit 800 |
| **BAF45A** | Proteintech | 66341-1-Ig | Clone No:  4D4C6 | Mouse  monoclonal | 1:1000 | Anti-Mouse680 |
| **BAF45A** | Thermo scientific | PA5-30678 | QK2110272E | Rabbit polyclonal | 1:1000 | Anti-Rabbit 800 |
| **BAF45D** | Santa Cruz Biotech | sc-514297 |  | Mouse monoclonal | 1:1000 | Anti-Rabbit 800 |
| **BRG1** | Santa Cruz Biotech | sc-17796 | K3016 | Mouse monoclonal | 1:500 | Anti-Mouse 680 |
| **BRD7** | Santa Cruz Biotech | sc-376180 | A0917 | Mouse monoclonal | 1:500 | Anti-Mouse 680 |
| **RUNX2** | Santa Cruz Biotech | sc-10758 | D1411 | Mouse monoclonal | 1:1000 | Anti-Mouse 680 |
| **GAPDH** | Santa Cruz Biotech | sc-32233 | H0411 | Mouse monoclonal | 1:500 | Anti-Mouse 680 |

# Supplementary Table 3: List of primary and secondary antibodies and their dilution used in this study
